# Obesity: An Overlooked Factor in Inflammatory Bowel Disease

**DOI:** 10.3390/ijms27093911

**Published:** 2026-04-28

**Authors:** Jiao Xie, Yan Ling, Shi Wang, Rui Gong

**Affiliations:** 1 Health Management Center, Union Hospital, Tongji Medical College, Huazhong University of Science and Technology, Wuhan 430022, China; 2 Department of Breast and Thyroid Surgery, Union Hospital, Tongji Medical College, Huazhong University of Science and Technology, Wuhan 430022, China

**Keywords:** obesity, inflammatory bowel disease, Crohn’s disease, ulcerative colitis

## Abstract

Inflammatory bowel disease (IBD) is a chronic, immune-mediated disorder characterized by relapsing inflammation of the gastrointestinal tract. Over the past decades, both the prevalence of IBD and the rates of obesity have increased globally, highlighting the potential impact of obesity on IBD pathogenesis and progression. Obesity is associated with a state of chronic low-grade inflammation, metabolic dysregulation, and altered gut microbiota, all of which may influence the onset, severity, and prognosis of IBD. However, existing evidence on the relationship between obesity and IBD remains limited and sometimes contradictory, with heterogeneity in study populations, obesity definitions, and outcome measures contributing to inconsistent findings. In this review, we summarize the epidemiology of obesity among IBD patients, explore the shared mechanisms linking obesity and IBD, discuss the influence of obesity on disease course, and evaluate the effects of currently available weight management interventions. This comprehensive overview aims to provide a clearer understanding of the complex interplay between obesity and IBD and to inform clinical management strategies.

## 1. Introduction

According to the World Health Organization (WHO), in 2022, approximately 2.5 billion adults (43%) worldwide were overweight (body mass index [BMI] ≥ 25 kg/m^2^), of whom 890 million (16%) were obese (BMI ≥ 30 kg/m^2^) [1]. In 2021, an estimated 129 million disability-adjusted life years (DALYs) across all age groups were attributed to obesity [2], and deaths associated with high BMI increased by more than 2.5-fold globally between 1990 and 2021 [3].

Inflammatory bowel disease (IBD) is a chronic, idiopathic disorder characterized by uncontrolled inflammation of the gastrointestinal (GI) tract, primarily comprising Crohn’s disease (CD) and ulcerative colitis (UC). Over the past decade, the incidence of IBD has increased globally, paralleling the rapidly rising prevalence of obesity [4,5,6]. The global crude prevalence of IBD increased from 3.32 million cases in 1990 to 4.9 million in 2019, while IBD-related deaths rose from 24,300 to 41,000 over the same period [7], imposing a substantial burden on healthcare systems. Although the underlying causes of the increasing incidence of IBD remain incompletely understood, it is widely recognized that multiple factors—including host genetics, dietary patterns, gut microbiota alterations, and environmental exposures—interact to drive inappropriate immune responses and mucosal injury [4,8,9,10]. In recent years, increasing attention has been directed toward the role of obesity in the pathogenesis and clinical course of IBD [11,12,13].

This review was conducted as a narrative review. A comprehensive literature search was performed in PubMed and Web of Science to identify relevant studies published up to January 2026. The following keywords and their combinations were used: “inflammatory bowel disease”, “Crohn’s disease”, “ulcerative colitis”, “obesity”, “overweight”, “body mass index”, “visceral adiposity”. Original research articles, cohort studies, clinical trials, and relevant review articles published in English were considered. Preclinical studies were included when necessary to support mechanistic insights but were interpreted with caution. Articles limited relevance to the topic were excluded. Given the narrative nature of this review, a formal quality assessment of included studies was not performed. However, priority was given to high-quality studies, including large cohort studies, meta-analyses, and well-designed clinical trials. This review summarizes the epidemiology of obesity in patients with IBD, explores the shared mechanisms underlying their interaction, evaluates the impact of obesity on disease course, and discusses current dietary, pharmacological, and surgical management strategies, aiming to provide a framework for optimizing clinical management.

## 2. Epidemiology

In recent years, the prevalence of obesity among patients with IBD has increased markedly, paralleling the global obesity epidemic. This trend is particularly evident in rapidly developing countries, such as China and India, and may be attributable to rising obesity rates and the westernization of lifestyle in these regions [14]. Current evidence suggests that obesity affects approximately 15–40% of patients with IBD [15,16,17], is more prevalent in Crohn’s disease (CD), and is associated with increased inflammatory activity [17]. The Crohn’s Disease Activity Index (CDAI) is a weighted composite score derived from stool frequency, abdominal pain, general well-being, extraintestinal complications, antidiarrheal use, abdominal mass, hematocrit, and body weight deviation [18]. Obese patients with CD exhibit higher CDAI scores and lower remission rates compared with their non-obese counterparts [19]. A 40-year cohort study in patients with UC reported a 2- to 3-fold increase in obesity prevalence and a significantly higher risk of hospitalization among obese individuals [20]. Moreover, obesity has been associated with longer hospital stays and increased rates of postoperative complications—including a 30–50% higher risk of wound infection and higher 30-day readmission rates [21,22]. In the United States, the prevalence of obesity among patients with IBD increased from 19.7% in 2010 to 30.1% in 2019, accompanied by a rising burden of metabolic syndrome (e.g., type 2 diabetes, hypertension, dyslipidemia) [19]. Cohort studies further indicate that higher BMI and increased waist circumference are positively associated with the risk of IBD, particularly CD, suggesting that obesity may contribute to disease development through chronic inflammation and metabolic dysregulation [23,24,25].

## 3. Mechanisms

**Figure 1:**
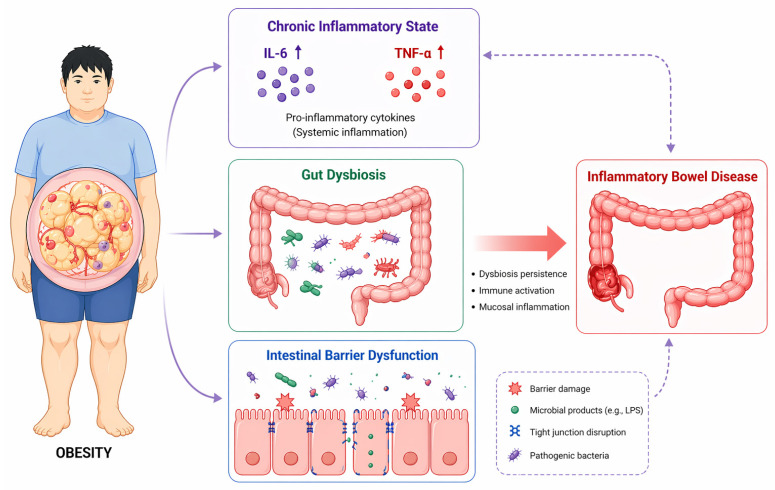
Schematic diagram illustrating the mechanisms linking obesity to IBD. Obesity is associated with gut dysbiosis, intestinal barrier dysfunction, and chronic inflammation, which together contribute to the development of IBD. Legend notes: Both solid arrows and dotted arrows indicate direct causal relationships between factors. Purple circles and red circles represent key inflammatory mediators involved in the pathological process of obesity-associated IBD.

The shared pathophysiological mechanisms underlying obesity and IBD include chronic inflammation, dysbiosis of the gut microbiota, and impaired intestinal barrier function (Figure 1).

### 3.1. Chronic Inflammatory State

Chronic inflammation associated with obesity is sustained by cytokines derived from white adipose tissue, which comprises both adipocytes and immune cells [26]. Interactions between adipocytes and immune cells modulate their secretory profiles, thereby influencing metabolic function and systemic inflammatory responses [27]. In obesity, adipocytes undergo hypertrophy and functional dysregulation, leading to increased secretion of adipokines and pro-inflammatory mediators, including tumor necrosis factor-α (TNF-α), interleukin-6 (IL-6), and leptin [28,29,30]. These mediators are also elevated in patients with active CD and contribute to systemic inflammation while influencing the intestinal microenvironment via the circulation. Notably, TNF-α and IL-6 serve not only as key markers of obesity-associated metabolic inflammation but also as central mediators of intestinal mucosal injury in IBD [31,32]. In addition, leptin levels are increased in obese individuals and further promote inflammatory and immune responses [33]. Collectively, these alterations contribute to a sustained pro-inflammatory state that may perpetuate immune-mediated diseases, including IBD [26]. Patients with CD frequently present with malnutrition. Leptin levels have been shown to be negatively correlated with nutritional risk scores, including the Nutritional Risk Screening 2002 (NRS-2002), which evaluates nutritional risk based on impaired nutritional status, disease severity, and age, with a total score ≥ 3 indicating nutritional risk [34], and the Patient-Generated Subjective Global Assessment (PG-SGA), which is a comprehensive nutritional assessment tool integrating weight loss, food intake, nutrition impact symptoms, functional status, metabolic stress, and physical examination, and it provides both a global nutritional category and a numerical triage score [35]. Conversely, leptin levels are positively associated with other nutritional indices, such as BMI, suggesting that leptin may influence the nutritional status of CD patients by regulating energy metabolism [36]. In a high-fat diet (HFD)-induced obese mouse model, elevated leptin levels, together with increased colonic leptin receptor expression and a marked reduction in pro-inflammatory cytokines, indicate that leptin may alleviate colitis via activation of anti-apoptotic pathways [37].

Moreover, the secretion of lipocalin, an adipokine with anti-inflammatory properties, is reduced in obese individuals. Lipocalin has been shown to counteract TNF-α mediated signaling pathways, including the nuclear factor kappa-B (NF-κB) pathway [38]. Consistently, lipocalin levels are negatively correlated with BMI and inflammatory markers such as IL-6 and TNF-α, suggesting a regulatory role in the metabolic–inflammatory axis of IBD [39,40]. In addition, obesity-associated insulin resistance and chronic hyperglycemia promote oxidative stress, enhance the activation of innate immune cells, and increase the release of pro-inflammatory mediators, thereby impairing intestinal epithelial repair capacity [41,42]. Emerging evidence highlights a strong association between mesenteric adipose tissue (MAT) and IBD, particularly in CD. In CD, MAT can wrap around inflamed intestinal segments, forming the characteristic “creeping fat” (CrF) during the inflammatory process [43]. MAT functions as an active endocrine organ, secreting a wide range of pro-inflammatory cytokines and adipocytokines, and serves as an important source of C-reactive protein (CRP) [44,45,46]. A case–control study demonstrated that the gene expression profile of visceral adipose tissue (VAT) in patients with CD is similar to that observed in obese individuals, suggesting that obese patients with CD may exhibit a more pronounced inflammatory state compared with their non-obese counterparts [31].

### 3.2. Gut Dysbiosis and Intestinal Barrier Dysfunction

Both patients with IBD and individuals with obesity exhibit reduced gut microbiota diversity. These alterations, together with compositional shifts in the microbiota, contribute to intestinal epithelial barrier dysfunction [47]. Specifically, dysbiosis is characterized by an altered *Bacteroidetes* to *Firmicutes* ratio and a marked reduction in short-chain fatty acids (SCFAs) producing bacteria, including butyrate-producing species such as *Roseburia* spp. [48,49]. Dysbiosis contributes to the reduced production of SCFAs, including butyrate and propionate, thereby disrupting energy metabolism and anti-inflammatory signaling pathways, while promoting the accumulation of pro-inflammatory metabolites such as lipopolysaccharide (LPS) [50]. In particular, the abundance of *Akkermansia muciniphila*, a mucin-degrading bacterium, is markedly reduced in both IBD and obesity. This depletion is closely associated with impaired intestinal barrier integrity and metabolic dysfunction [51]. Consistent with these observations, in vitro studies demonstrate that *Akkermansia muciniphila* adheres to intestinal epithelial cells and enhances epithelial barrier function, supporting its protective role in maintaining gut homeostasis under obese conditions [52].

Furthermore, in a high-fat-diet-induced model of intestinal dysbiosis, extracellular vesicles derived from *Akkermansia muciniphila* (AmEVs) have been shown to improve intestinal mucosal barrier function by enhancing epithelial tight junction protein expression, increasing IL-10 levels, and reducing inflammatory markers in the colon. In addition, AmEVs have been shown to decrease intestinal permeability and modulate immune signaling by inhibiting Toll-like receptor-4 (TLR-4) and interferon-α (IFN-α) expression via AMP-activated protein kinase (AMPK), while promoting TLR2 expression and IL-4 production in Caco-2 cells in vitro [53,54]. Obesity-associated dysbiosis may impair intestinal barrier integrity, leading to increased bacterial translocation and subsequent metabolic endotoxemia. This process may further activate innate immune pathways, including the NOD-like receptor pyrin domain-containing 3 (NLRP3) inflammasome, thereby amplifying intestinal inflammation in IBD [55]. Taken together, these findings suggest that components of *Akkermansia muciniphila*, particularly its extracellular vesicles, may represent potential therapeutic targets in IBD.

Importantly, many of the mechanistic insights described above are derived primarily from experimental models, including animal studies and in vitro systems. While these findings provide valuable biological plausibility, their direct clinical relevance in human IBD remains to be fully established. Therefore, caution should be exercised when extrapolating these results to clinical practice, and further well-designed human studies are required to validate these potential mechanisms.

### 3.3. Clinical Impact of Obesity on IBD

Emerging evidence suggests that visceral adiposity, a more metabolically active tissue compartment than BMI, may play a greater role in driving inflammatory processes in IBD [56]. However, the overall impact of obesity on IBD outcomes remains limited and inconclusive. Clinical studies indicate that obese patients with IBD tend to exhibit higher disease activity, particularly in CD, where obesity is associated with increased CDAI scores and lower remission rates. In contrast, the association between obesity and disease activity appears to be weaker in UC [57]. Evidence regarding the impact of obesity on clinical outcomes in IBD remains inconsistent. Some studies have reported that obese patients are less likely to receive anti-tumor necrosis factor (anti-TNF) therapy, undergo surgery, or require hospitalization [15], and that obesity does not adversely affect long-term disease progression [16]. In contrast, large-scale cohort data indicate that obesity is associated with higher risks of 30- and 90-day readmission, prolonged hospital stays [22] and increased corticosteroid use [20]. Furthermore, obesity has been linked to reduced efficacy of several advanced therapies, including TNF-α inhibitors, vedolizumab, ustekinumab, and Janus kinase inhibitors, particularly in patients with UC [58]. A systematic review found no significant differences in corticosteroid use, hospitalization, surgical intervention, or emergency department visits between obese and normal-weight patients with IBD [59]. Similarly, a recent multicenter study of IBD patients receiving biologic therapy reported no association between obesity and the risks of hospitalization, surgery, or serious infections [11]. Therefore, further studies are needed to clarify the impact of obesity on the clinical course of IBD. The key epidemiological and clinical findings discussed above are summarized in Table 1. 

Several potential sources of heterogeneity may account for these discrepancies. First, differences in the definition and assessment of obesity represent a major source of variability. Most studies rely on BMI; however, BMI has several important limitations. Notably, BMI fails to identify sarcopenia, and a normal body weight does not necessarily indicate adequate nutritional status. A study published in 2017 demonstrated that “overweight but sarcopenic” phenotypes are not uncommon in patients with IBD [60], highlighting the importance of assessing muscle mass in this population. In addition, BMI does not reflect fat distribution, particularly visceral adiposity. Several studies have shown that visceral fat is a stronger predictor of adverse outcomes than BMI alone [61,62,63]. Therefore, reliance solely on BMI may lead to underdiagnosis of sarcopenia, sarcopenic obesity, and occult malnutrition. Nevertheless, BMI remains advantageous due to its simplicity, low cost, and suitability for longitudinal monitoring and initial screening. Accordingly, the optimal clinical approach is not to abandon BMI, but to use it as a preliminary assessment tool in combination with body composition analysis and standardized nutritional screening instruments. Second, intrinsic disease heterogeneity between CD and UC also contributes to inconsistent findings. CD is more frequently associated with mesenteric fat expansion, whereas the relationship between obesity and UC appears to be less pronounced. Third, differences in outcome measures across studies further complicate interpretation. Various studies have evaluated disease activity, hospitalization risk, surgical outcomes, and therapeutic response, and these endpoints are not directly comparable. Fourth, treatment-related factors may influence study outcomes. Obesity has been reported to alter the pharmacokinetics of biologic agents, potentially affecting treatment efficacy [64]. Fifth, differences in body composition, including sarcopenia and sarcopenic obesity, may further confound results. These conditions have been associated with worse clinical outcomes, independent of BMI [60]. In summary, these factors highlight the complexity of the relationship between obesity and IBD and underscore the need for standardized definitions and well-designed prospective studies.

## 4. Impact of Obesity Interventions on IBD

Despite the rising prevalence of obesity among patients with IBD, only a small proportion undergo weight management interventions [19]. A large-scale study including more than 39 million patients between 2010 and 2019 reported that, although approximately 37.3% of IBD patients were obese, only 2.8% received pharmacological treatment [19]. Current management strategies for obesity include dietary interventions, anti-obesity medications (AOMs) and bariatric surgery.

### 4.1. Dietary Interventions

Dietary interventions are considered a first-line strategy for the management of obesity. Various dietary approaches have been shown to modulate disease activity and clinical outcomes in IBD. This review focuses on commonly recommended dietary patterns, including the Mediterranean diet (MD), ketogenic diet (KD), specific carbohydrate diet (SCD), anti-inflammatory diet, and the low fermentable oligosaccharides, disaccharides, monosaccharides, and polyols (low-FODMAP) diet.

#### 4.1.1. MD

The MD, characterized by a high intake of vegetables, fruits, whole grains, and olive oil [65,66], has been widely associated with multiple health benefits, including anti-inflammatory effects and protection against chronic diseases such as colorectal cancer [67]. Given that IBD is characterized by chronic inflammation and gut microbiota dysbiosis, emerging evidence suggests that the MD may help restore intestinal microbial balance by promoting the abundance of anti-inflammatory bacterial species. In addition, the MD has been shown to reduce inflammatory markers in patients with IBD, potentially through its bioactive components, including omega-3 fatty acids, polyphenols, and dietary fiber [68,69]. The anti-inflammatory effects of the Mediterranean diet are largely attributed to its key bioactive components. Omega-3 fatty acids have been shown to modulate inflammatory signaling pathways, including inhibition of NF-κB, reduce the production of pro-inflammatory cytokines such as TNF-α and IL-6, and promote the biosynthesis of specialized pro-resolving mediators, which contribute to the resolution of inflammation [70,71]. Polyphenolic compounds possess antioxidant and immunomodulatory properties, attenuating oxidative stress, suppressing inflammatory signaling pathways, and modulating gut microbiota composition and host immune responses [72,73]. Dietary fiber contributes to intestinal homeostasis through microbial fermentation and the production of SCFAs, particularly butyrate, which enhance epithelial barrier integrity, regulate immune responses, and inhibit intestinal inflammation [74,75]. In recent years, increasing evidence has highlighted the beneficial effects of the MD in patients with IBD. The MD has been associated with improvements in obesity-related parameters, including reductions in BMI, waist circumference, and hepatic fat accumulation, as well as modulation of fecal calprotectin levels, a key marker of disease activity in UC [76,77]. In addition, the MD has been shown to improve nutritional status, reduce the accumulation of pro-inflammatory visceral adipose tissue [78] and lower the prevalence of metabolic syndrome in patients with IBD [79].

#### 4.1.2. KD

The KD, characterized by high fat, low carbohydrate, and moderate protein intake, has recently emerged as a potential therapeutic approach for IBD. The KD promotes the production of ketone bodies, particularly β-hydroxybutyrate, by restricting carbohydrate intake and enhancing fatty acid metabolism. These metabolites exert anti-inflammatory effects by suppressing the release of pro-inflammatory cytokines, including IL-6, IL-18, IL-22, and TNF-α, thereby alleviating intestinal inflammation [80,81]. Mechanistically, ketone bodies may further attenuate intestinal inflammation through modulation of key signaling pathways, such as NF-κB and NLRP3 inflammasome [82]. Preclinical studies have demonstrated that a ketogenic diet attenuates disease activity in dextran sulfate sodium (DSS)-induced colitis models [83]. Case reports have described improvements in clinical symptoms and quality of life in patients with IBD following ketogenic or similar dietary patterns (e.g., meat-based diets). Notably, these interventions have been associated with substantial increases in Inflammatory Bowel Disease Questionnaire (IBDQ) scores, along with reported adherence to diets primarily composed of meat, eggs, and animal fats [81]. Evidence indicates that the KD can upregulate the expression of tight junction proteins and enhance intestinal mucosal integrity [80,84]. However, conflicting findings have been reported, with some studies suggesting that the KD may increase intestinal permeability, particularly in DSS-induced colitis models, indicating that the source and composition of dietary fat may influence its effects [81,85]. Current evidence supports the use of the KD as a potential adjunctive therapeutic strategy; however, its implementation should be individualized and conducted under medical supervision, given concerns regarding metabolic side effects. Further well-designed clinical studies are warranted to clarify its long-term safety and to identify patient populations most likely to benefit.

#### 4.1.3. SCD

The SCD is a restrictive dietary approach that eliminates foods containing complex or poorly absorbed carbohydrates. It is based on the premise that reducing the fermentation of unabsorbed carbohydrates in the gut can inhibit pathogenic bacterial overgrowth, decrease inflammatory responses, and potentially improve the disease course of IBD by modulating the intestinal microbiota [86,87]. Several studies have demonstrated that the SCD is effective in improving symptoms during the active phase of CD [88]. In addition, SCD intervention has been associated with significant reductions in inflammatory markers, including CRP and erythrocyte sedimentation rate (ESR), in patients with IBD [89], but its long-term effects on mucosal healing remain unclear [90]. Moreover, the restrictive nature of the diet may increase the risk of insufficient caloric intake and unintended weight loss, as it limits the consumption of complex carbohydrates, which may also negatively affect long-term adherence. In a study including 417 patients with IBD, remission was achieved at different time points following SCD intervention, with 13% of patients achieving remission within 2 weeks, 17% between 2 weeks and 1 month, 36% between 1 and 3 months, and 34% after more than 3 months [88]. However, these findings should be interpreted with caution, as 56% of participants received concomitant medical therapy, which may have introduced treatment-related bias. In pediatric patients with IBD, SCD has also been associated with significant alterations in the fecal microbiome, including increased abundance of beneficial bacteria and reductions in inflammatory markers [89,91,92]. Despite these potential benefits, SCD may not be suitable for all patients and should be individualized based on disease phenotype, activity, and nutritional status [93].

#### 4.1.4. Anti-Inflammatory Diet

Evidence indicates that the Dietary Inflammation Index (DII) is positively associated with disease activity in patients with CD, with significantly higher scores observed in patients with mild to moderate active disease compared with those in remission. In contrast, no significant association has been reported in patients with UC [94]. Long-term adherence to an anti-inflammatory diet may reduce the risk of developing IBD. In contrast, Western dietary patterns, characterized by high fat and low fiber intake, are associated with an increased incidence of IBD. These protective effects of anti-inflammatory diets may be mediated through the reduction of oxidative stress and modulation of immune dysregulation [95]. Anti-inflammatory dietary patterns may attenuate intestinal inflammation by reducing pro-inflammatory mediators and enhancing anti-inflammatory components. An interventional study demonstrated that patients with IBD who followed an anti-inflammatory diet characterized by reduced intake of red meat, fried foods, and high-lactose products achieved higher rates of sustained clinical remission [96]. Mechanistically, dietary fiber and its fermentation products support intestinal barrier integrity by promoting the growth of beneficial bacteria, such as Bifidobacterium, while inhibiting pathogenic species [97]. In addition, polyphenols exert anti-inflammatory effects by enhancing mucus secretion and tight junction function, increasing antioxidant enzyme activity, suppressing inflammatory signaling pathways and cytokine production, and modulating the gut microbiota toward a more favorable composition, including enrichment of probiotic and SCFAs producing bacteria [98]. Overall, anti-inflammatory diets show promise in the management of IBD through multiple mechanisms, including suppression of inflammation, modulation of immune responses, and restoration of microbial balance. However, their clinical application should be individualized. Future research should focus on the development of standardized dietary protocols, identification of predictive biomarkers, and evaluation of synergistic effects with other therapeutic strategies.

#### 4.1.5. Low FODMAP Diet

The low FODMAP diet, which restricts the intake of fermentable oligosaccharides, disaccharides, monosaccharides, and polyols, is primarily used in the management of IBD patients with coexisting irritable bowel syndrome and in those in clinical remission. This dietary approach is typically implemented in three phases, including restriction, reintroduction, and personalization [99]. It is generally recommended for patients without active intestinal inflammation, as its effects on the gut microbiota may be unfavorable in the context of active disease [100,101]. Approximately 30 percent of patients with IBD in remission meet the diagnostic criteria for irritable bowel syndrome, and evidence suggests that this dietary approach significantly improves symptoms such as bloating and diarrhea in this population [102]. Mechanistically, by reducing intestinal osmotic load and luminal distension, this diet can alleviate gastrointestinal symptoms, including diarrhea and constipation, particularly in patients with IBD in remission who present with IBS-like symptoms [103]. FODMAP carbohydrates are poorly absorbed in the small intestine and are readily fermented by colonic bacteria, leading to gas production and symptoms such as bloating and diarrhea [104]. By restricting these substrates, the low FODMAP diet can alleviate gastrointestinal symptoms. However, it may also reduce the overall abundance of gut microbiota, including beneficial species such as Bifidobacterium [105]. Given that Bifidobacterium plays an important role in immune regulation and has anti-inflammatory and potential anticancer properties, this reduction raises concerns regarding the long-term impact of the diet on gut health [106]. Therefore, the low FODMAP diet is generally recommended as a short-term strategy for symptom control, followed by gradual reintroduction of high FODMAP foods to minimize potential adverse effects on the gut microbiota [107].

### 4.2. Anti-Obesity Medication (AOM), Bariatric Surgery

Anti-obesity medications represent a promising therapeutic option for patients with IBD and coexisting obesity. Currently available pharmacological agents include orlistat, liraglutide, phentermine topiramate, and naltrexone bupropion [108]. Among these, glucagon-like peptide-1 (GLP-1) receptor agonists, such as liraglutide, have demonstrated efficacy in reducing body weight and appear to be safe for use in patients with IBD without exacerbating disease activity [109,110,111]. In patients with concomitant diabetes, treatment with GLP-1 receptor agonists has also been associated with a reduced risk of surgery [112]. Mechanistically, GLP-1 receptor agonists may exert anti-inflammatory effects by modulating the gut microbiota. This includes increasing the abundance of beneficial bacteria, such as *Lactobacillus roissyi*, and reducing pathogenic species such as *Staphylococcus intestinalis*, potentially through regulation of the microbiota DMS IL-22-positive ILC3 axis [113]. GLP-1 receptor agonists have attracted increasing attention in the context of obesity and IBD. Current evidence suggests that these agents are generally safe and may provide metabolic benefits in patients with coexisting obesity and IBD. However, there is currently insufficient evidence to support the idea of a direct anti-inflammatory effect of GLP-1 receptor agonists as a primary treatment for IBD. Most available data are derived from observational studies and focus on metabolic outcomes rather than disease modification. Therefore, while GLP-1 receptor agonists may represent a promising adjunctive strategy in selected patients, their role in IBD management remains to be fully established. Bariatric surgery represents an effective strategy for weight loss in patients with obesity, including those with IBD. The most commonly performed procedures include laparoscopic adjustable gastric banding (LAGB), Roux-en-Y gastric bypass (RYGB), and sleeve gastrectomy (SG) [114,115]. Current evidence suggests that bariatric surgery is generally safe and effective in patients with IBD, with postoperative complication rates comparable to those observed in the general population, along with substantial weight reduction at 6 and 12 months after surgery [116]. However, emerging data indicate a potential association between bariatric surgery and the development of new onset IBD, particularly CD. This risk may be related to alterations in intestinal anatomy, changes in nutrient absorption, and disruption of the gut microbiota. Notably, RYGB has been associated with a higher risk of CD, whereas sleeve gastrectomy appears to be more frequently linked to UC [116,117]. The specific biological and clinical effects of obesity-related interventions in patients with IBD are summarized in Table 2.

## 5. Conclusions

In conclusion, the relationship between obesity and IBD is complex and multifactorial, with growing evidence supporting both epidemiological associations and underlying biological mechanisms.

Several aspects are relatively well established. The prevalence of obesity among patients with IBD continues to rise and is linked to chronic low-grade inflammation, alterations in the gut microbiota, and impairment of intestinal barrier function. Importantly, measures of body composition, such as visceral adiposity and sarcopenia, appear to provide more clinically relevant information than BMI alone. However, significant uncertainties remain. The impact of obesity on disease activity, therapeutic response, and long-term outcomes in IBD remains inconsistent across studies. These discrepancies likely reflect heterogeneity in study design, definitions of obesity, disease phenotypes, and outcome measures. In addition, the clinical relevance of mechanistic insights derived from experimental models, including those involving adipokines and microbiota-related pathways, has yet to be fully established.

Future research should prioritize the development of standardized definitions of obesity in IBD, broader application of body composition assessment, and well-designed prospective studies to clarify causal relationships. Translational research will also be essential to bridge the gap between experimental findings and clinical application. From a clinical perspective, several implications can already be considered. While BMI remains a useful initial screening tool, it should be complemented by more precise assessments of body composition and nutritional status. Clinicians should also consider the potential influence of obesity on pharmacokinetics and treatment response and adopt individualized management strategies for patients with coexisting obesity and IBD. Overall, a more refined and standardized framework for evaluating obesity in IBD is essential to advance both clinical practice and future research.

## Figures and Tables

**Table 1:** Clinical studies evaluating the impact of obesity on IBD outcomes.

Study	Study Design	Population (*N*)	Outcomes Assessed	Key Findings
Greuter et al. [57]	Retrospective	IBD:*N* = 2120	Disease activity, remission, disease course	Obese patients with CD exhibit higher disease activity, lower remission rates, and an increased risk of a complicated disease course; however, obesity is not associated with long-term disease progression
Flores, A. et al. [15]	Retrospective	IBD:*N* = 581	Treatment, surgery, hospitalization	Obesity in patients with IBD is associated with a lower likelihood of receiving anti-TNF therapy, undergoing surgery, or requiring hospitalization
Weissman, S. et al. [22]	Retrospective	IBD:*N* = 143,190	Readmission, hospital stay, cost	Obesity in IBD is independently associated with higher readmission rates, longer hospital stays, and increased healthcare costs
Johnson, A.M. et al. [20]	Retrospective	UC:*N* = 417	Hospitalization, steroid use	Obesity is associated with an increased risk of hospitalization and corticosteroid use in patients with IBD
Desai, A. et al. [58]	Retrospective	UC:*N* = 18,696	Treatment response	Obesity is associated with lower efficacy of several advanced therapies in patients with UC
Seminerio, J.L. et al. [59]	Retrospective	IBD:*N* = 1494	Prednisone use, emergency, hospitalization, surgery	Increasing BMI is not associated with clinical outcomes
Gu, P. et al. [11]	Prospective	IBD:*N* = 3038	Hospitalization, surgery, infections	Obesity is not associated with the risk of hospitalization, surgery, or serious infections

**Table 2:** Effects of obesity-related interventions in IBD.

Study	Study Design	Intervention	Population	Biological Indicators	Clinical Outcomes
[65,66,67,68,69,70,71,72,73,74,75,76,77,78,79]	Clinical studies	MD	IBD patients	BMI ↓, liver fat ↓, waist circumference ↓, fecal calprotectin ↓, microbiota composition improved	Metabolic status improved, reduced risk of metabolic syndrome, decreased relapse risk in CD
[80,81,82,83,84,85]	Animal studies, case reports	KD	Animal models, IBD patients	Body weight ↓, TNF-α, IL-6, IL-18 ↓, tight junction proteins ↑, barrier function improved	Disease activity ↓, IBDQ score ↑
[86,87,88,89,90,91,92,93]	Cohort, clinical studies	SCD	CD, IBD patients	Nutritional status improved; CRP, ESR ↓, beneficial bacteria ↑	Remission achieved in some patients, symptoms improved
[94,95,96,97,98]	Observational, interventional studies	Anti-inflammatory diet	IBD patients	Inflammatory response ↓, SCFAs ↑, beneficial bacteria ↑; barrier function improved	Remission maintenance ↑, disease activity ↓
[99,100,101,102,103,104,105,106,107]	Clinical, observational studies	Low-FODMAP diet	IBD, IBS patients	Total microbiota ↓	Improves bloating, diarrhea
[108,109,110,111,112,113]	Clinical, experimental studies	GLP-1 receptor agonists	Obese IBD patients	Body weight ↓, beneficial bacteria ↑, pathogenic bacteria ↓	Good safety profile, reduced surgical risk
[114,115,116,117]	Cohort studies	Bariatric surgery	Obese IBD patients	BMI ↓, CRP ↓	Significant weight loss, possible increased risk of incident IBD

Notes: ↑ indicates an increase, and ↓ indicates a decrease in the corresponding parameter after the intervention.

## Data Availability

No new data were created or analyzed in this study. Data sharing is not applicable to this article.

## Abbreviations

The following abbreviations are used in this manuscript:

AmEVs	*Akkermansia muciniphila*-derived extracellular vesicles
AMPK	AMP-activated protein kinase
AOM	Anti-obesity medications
BMI	Body Mass Index
CD	Crohn’s disease
CDAI	Crohn’s Disease Activity Index
CrF	Creeping fat
CRP	C-reactive protein
DII	Dietary Inflammation Index
DSS	Dextran sulfate sodium
ESR	Erythrocyte sedimentation rate
GI	Gastrointestinal
GLP-1	Glucagon-like peptide-1
HFD	High-fat diet
IFN-α	Interferon-α
IL-6	Interleukin-6
IBS	Irritable bowel syndrome
IBDQ	Inflammatory Bowel Disease Questionnaire
IBD	Inflammatory bowel disease
KD	Ketogenic diet
LAGB	Laparoscopic adjustable gastric banding
LPS	Lipopolysaccharide
MD	Mediterranean diet
MAT	Mesenteric adipose tissue
RYGB	Roux-en-Y gastric bypass
SCFAs	Short-chain fatty acids
SCD	Specific carbohydrate diet
SG	Sleeve gastrectomy
TLR-4	Toll-like receptor-4
TNF-α	Tumor necrosis factor-alpha
UC	Ulcerative colitis
VAT	Visceral adipose tissue
WHO	World Health Organization
